# Changes in values and well-being amidst the COVID-19 pandemic in Poland

**DOI:** 10.1371/journal.pone.0255491

**Published:** 2021-09-15

**Authors:** Agnieszka Bojanowska, Łukasz D. Kaczmarek, Maciej Koscielniak, Beata Urbańska

**Affiliations:** 1 Institute of Psychology, SWPS University of Social Sciences and Humanities, Warsaw, Poland; 2 Department of Psychology and Cognitive Science, Adam Mickiewicz University, Poznan, Poland; Sciensano, BELGIUM

## Abstract

COVID-19 caused a global change in the lifestyles of people around the world. It provided a unique opportunity to examine how external circumstances impact two crucial aspects of functioning relating to "who I am" (values) and "how I feel" (well-being). Participants (*N* = 215) reported their values and subjective and eudaimonic well-being, nine months before the first lockdown in Poland and two weeks and four weeks into the first lockdown. We observed increased valuing of self-direction, security, conformity, humility, caring, and universalism and a decrease in valuing hedonism. Individuals experienced decreased subjective and eudaimonic well-being, with women responding with stronger negative affect intensity relative to men. Finally, we identified that individuals who were more open to change before the COVID-19 pandemic responded with higher eudaimonic well-being two weeks into lockdown relative to their less open to change peers. This study is unique in that it shows that well-being and individually held values are flexible and adaptive systems that react to external circumstances such as global critical events.

## Introduction

The dynamic of well-being and individual value hierarchies have been the focus of psychological research for several decades. These two individual differences reflect significant aspects of human psychological functioning and are intertwined [1]. Furthermore, there is evidence suggesting that, to some extent, well-being and value changes reflect external circumstances [2, 3]. The COVID-19 lockdown restrictions introduced in many countries to prevent the spread of the virus provided a unique opportunity to study the impact of a severe global threat on people’s psychological functioning. Understanding possible changes in well-being and values related to pandemics is essential in making informed decisions on health-protective, behavioral and environmental restrictions and support during the COVID-19 and future similar pandemics. In the present study, we aimed to examine how the COVID-19 pandemic was related to changes in values [4], subjective well-being [5], and eudaimonic well-being [6]. We present how personal values and well-being changed two and four weeks into the lockdown-like restrictions introduced in Poland on March 14th, 2020, relative to nine months earlier.

### Stability and change of eudaimonic and hedonic well-being

Well-being is best conceptualized as a compound of hedonia and eudaimonia–two related dimensions [7]. Hedonic well-being is represented by the subjective well-being theory accounting for the cognitive and affective components [5]. The cognitive component pertains to satisfaction with life, i.e., an individual’s general belief about their life as similar to or different from their subjective ideal. The affective component emphasizes the abundance of positive emotions and the absence of negative ones in daily life. Eudaimonic well-being represents the realization and expression of one’s human and individual potential [6]. From this perspective, well-being is a process of fulfilling a person’s "daimon," or true nature, through self-discovery, a sense of purpose and meaning in life, intense involvement in activities, and investment of significant effort.

Examining the extent to which well-being is influenced by severe worldwide threats, such as the COVID-19 pandemic, is important because, contrary to several previous studies and theories advocating for the stability of well-being, comprehensive data suggests otherwise. Twin studies supported well-being stability indicating that genetic influence rather than circumstances explain up to 52% of the variance in subjective well-being. Other factors had a negligible effect after controlling for the genetically determined happiness level [8]. Other studies suggested that even dramatic social events such as a national economic downfall can have little-to-no impact on people’s satisfaction with life [9]. The stability of well-being in the face of adverse events is explained by the adaptation-level theory of well-being [10], personality theory of subjective well-being [11], or the dynamic equilibrium theory [12].

However, these stability theories have been subject to criticism because prolonged circumstances such as unemployment (negative influence) or marriage (positive influence) can substantially and semi-permanently change individuals’ satisfaction with life baseline [13, 14]. Moreover, another group of theories and research suggests that well-being has high temporal instability [3]. For instance, short-term or occasion-specific satisfaction levels with life can depend on people’s intentional activity, i.e., what individuals choose to do [15, 16]. In addition, several studies have documented that changes in behavior can temporarily influence well-being [17–21]. Finally, there is some evidence of gender differences in baseline well-being. Women tend to experience lower satisfaction with life and higher negative affect (data on gender differences in positive affect is inconclusive) [22]. Inferences concerning gender differences in well-being stability can be drawn from the personality theory of subjective well-being [11]. This theory links well-being with neuroticism, which is responsible for emotional reactivity. Since women score higher on neuroticism [23] their affective reactions to adverse events may be stronger than among men, especially in negative affect.

Studies indicate that the COVID-19 pandemic caused a significant decrease in well-being in China [24, 25], the USA [26], Japan [27], Ireland [28], and New Zealand [29]. Thus, we expected that the adverse effects of the COVID-19 pandemic on well-being would also replicate in Poland. However, previous studies focused on specific aspects of well-being (primarily emotional or subjective well-being). Therefore, we aimed to extend these findings by also including eudaimonic well-being.

### Human values: Their structure, functions, and relationship with well-being

Values determine the things that people consider to be important and worthwhile [4, 30]. They guide intentional behavior and motivate people to pursue ideas and activities consistent with their values and to avoid those that go against them [31–34]. The structure of values takes a circular form. Similar values that lie next to one another on the circle are complementary (e.g., conformity and tradition) and can be realized by one activity. In contrast, those that lie on the opposite parts of the circle stand in opposition to each other (e.g., stimulation vs. security) and cannot be realized simultaneously. Consequently, values impact the way people behave. This may be vital for the spread of the virus and how people behave towards one another in a time of crisis, e.g., through engaging in helpful acts, donating money to charity, or volunteering [35–37].

The circular structure of values also allows for the grouping of values into higher-order dimensions: 1) Openness to change: self-direction thought, self-direction action, stimulation, hedonism; 2) Self-enhancement: achievement, power-dominance, power-resources; 3) Conservation: humility, conformity-interpersonal, conformity-rules, tradition, security-personal, security-societal, face; and 4) Self-transcendence: universalism-tolerance, universalism-nature, universalism-concern, benevolence-caring, benevolence-dependability. Grouping such values into higher-order dimensions is an expression of the patterns of conflict and congruity between them, and each dimension expresses a specific set of adjacent values [4].

Values and well-being can be associated directly or indirectly [38]. Each value is related to specific attitudes and beliefs about the world. This may impact well-being directly (e.g., people valuing benevolence believe that people are friendly and tend to be tolerant, which leads to enhanced well-being). Indirectly, values may impact well-being through different activities chosen by people holding different values (e.g., engagement in pleasure-seeking among those valuing hedonism). Studies on specific links between value dimensions and well-being have not yet provided definitive answers [38]. However, the above associations suggest that some values may be favorable to well-being, while others may be unfavorable. Further, the above functions of values may serve as a protective force, shielding from well-being changes in the face of adverse events. More data from different contexts are needed to identify, for example, possible situational moderators of the value and well-being relationship. The COVID-19 pandemic provided a unique opportunity and may help answer questions about the function of values for well-being in adverse circumstances.

### Change in values and circumstances

Values will likely change in response to external circumstances because their function is to channel energy and skills to effectively cope with upcoming challenges and normative forces [39]. Furthermore, negative situations result in changes towards focusing on security, while positive changes bring attention to self-expression [2]. Evidence from studies on national or global crises supported these claims about Schwartz’s value theory [4]. These studies also indicated that changes in any given value are accompanied by a change in the opposing values in the circle [40]. For example, the worldwide financial crisis in 2008 impacted the values of youth and young adults; specifically, values such as security, tradition, benevolence, and conformity significantly increased, while the importance of hedonism, self-direction, and stimulation decreased [40]. Verkasalo, Goodwin, and Bezmenova observed an increase in security values and a decrease in stimulation values within a few days of the World Trade Center attack [41]. In a study on Israeli adolescents during and after the Israeli-Lebanese war, the importance placed upon tradition, power, and security values increased. At the same time, benevolence, universalism, self-direction, stimulation, and hedonism decreased [42]. The current paper aims to replicate similar outcomes by examining the impact of the COVID-19 pandemic crisis on the structure of human values. To the best of our knowledge, no research investigated COVID-19-related changes in the structure of personal values.

### The course of the COVID-19 pandemic in Poland

The Polish government responded to the COVID-19 crisis after dramatic events unfolded in Italy, where the infection growth curve significantly hampered the effectiveness of the healthcare system. Consequently, strict preventive measures that changed the everyday functioning of the nation were introduced, such as closing the borders, shutting down schools, introducing quarantine for people coming from abroad, and business shutdown. This resulted in a relatively slow-growing curve of infections observed four weeks after the lockdown (https://covid19.who.int/). However, the first two weeks were dominated by uncertainty and doubts about whether the Polish healthcare system would provide adequate service if the rate of infections was as high as in Italy. The first lockdown-type measures were introduced on March 13th and continued to be tightened well into April (www.gov.pl). This included restrictions in international travel, mass events, access to public parks and forests, and the closure of schools, universities, restaurants, beauty salons, shopping malls, etc. On April 20^th^, the government began to ease these restrictions, first by allowing citizens to enter parks and forests. This timeline is essential for the present article: the middle measurement was taken on March 29^th^, 2020, about two weeks after the first restrictions had been introduced, and the third two weeks later, on April 12^th^, 2020. The first baseline measurement was taken nine months prior.

During this time, the number of reported COVID-19 infections and related deaths in Poland were 68 cases up to 13th March 2020 (2 deaths), 1,864 cases up to 29th March (22 deaths) and 6,674 up to April 12^th^, 2020 (232 deaths; https://www.worldometers.info/coronavirus/country/poland/). These numbers may seem small, but the context in which Poles functioned at the time included intense daily media coverage of the situation in Italy, in which there were 18,000 infections (1,266 total confirmed deceased) on 13th March 2020, almost 98,000 infections (almost 11,000 total deaths) on March 29th and 156,362 (19,899 total deaths) on April 12^th^, 2020 (https://www.worldometers.info/coronavirus/country/italy/). Similar data were also emerging from Spain (5,232 infections until March 13^th^, 2020, total deaths; 133; 80,110 infections until March 29^th^, 2020, total deaths 6,803; 166,831infections until April 12^th^, 2020, total deaths 17,209) and other countries. This was accompanied by numerous articles voicing concern about the state of public health services in Poland and confirmed by a public opinion survey conducted at the time, in which 62% of Poles stated that they were scared that they would contract the virus and only 32% were convinced that the public health services were prepared to handle the pandemic (https://www.cbos.pl/SPISKOM.POL/2020/K_040_20.PDF).

### Present study

This study aimed to examine how well-being and values changed during the COVID-19 pandemic in Poland. We expected a decrease in hedonic values and an increase in security and tradition, and other prosocial values. In addition, building upon the basic needs theory [4], well-being models [5, 6], and findings from other countries during this and previous crises, we expected that individuals would respond with decreased well-being to the COVID-19 pandemic and this change would be moderated by gender. Finally, we built upon the links between values and well-being, investigating whether individuals with higher pre-pandemic levels of specific values were more likely to present a healthier response to the pandemic marked by a less severe change in their well-being two weeks into lockdown.

## Methods

### Participants

The participants were *N* = 215 Polish adults (58.10% women) aged between 22 and 79 (*M* = 54.66; *SD* = 13.25), drawn from an initial pool of *N* = 1,161 Polish adults (55% women) aged between 18 and 78 (*M* = 45.00, *SD* = 15.01). The initial pool represented our ongoing longitudinal project on values and well-being, which started before the COVID-19 pandemic. The study was conducted online via a professional research panel. The data in the first measurement were collected from a national sample representative of gender, age, education, and Polish regions. We took the first measurement (T1) in June 2019, i.e., nine months before the first COVID-19 lockdown in Poland. The second measurement (T2) was taken two weeks into the first lockdown, and the third (T3) four weeks into the lockdown.

The panel approached all participants from the initial pool, and from that sample, *N* = 227 completed the measurement in T2 and T3. We excluded N = 12 participants from the final pool due to unreasonably short response times and a lack of small variance between their answers. The participants received points for their participation, which they could then exchange for small gifts. Each participant provided written informed consent. The study was approved by the SWPS University Ethics Committee (opinions no. 20/2019 for T1, 26/2020, for T2 and T3).

### Measures

#### Satisfaction with life

We used the Diener’s Satisfaction with Life Scale [43, 44] to assess participants’ global evaluation of their life (’In most ways, my life is close to my ideal’) on a scale from 1 (’I definitely disagree’) to 7 (’I definitely agree’). This is a five-item scale (one dimension). Higher scores represent higher satisfaction with life. The scale had high internal consistency: Cronbach’s alphas and McDonald’s omegas were above 0.90 for all measurements.

#### Positive and negative affect

Positive and negative affect were measured using the Positive and Negative Affect Schedule by Watson, Clark, and Tellegen [45, 46]. This measure contains a list of ten adjectives referring to positive (e.g., interested, excited) and ten referring to negative (e.g., guilty, ashamed) affective states experienced over the previous two weeks. Participants responded on a scale from 1 (’slightly or not at all’) to 5 (’extremely’). Higher scores represent a higher intensity of each affective dimension. Cronbach’s alphas and McDonald’s omegas were above 0.78 for all measurements.

#### Eudaimonic well-being

We used Waterman’s Questionnaire for Eudaimonic Well-being [6, 47], which consists of 21 items (e.g., ’I believe I know what my strongest skills are and I try to develop them whenever possible’), with answers ranging from 1 (’strongly agree’) to 7 (’strongly disagree’). The general dimension of eudaimonic well-being is calculated using this tool. Higher scores indicate higher eudaimonic well-being. Cronbach’s alphas and McDonald’s omegas were above 0.87 for all measurements.

#### Values

Schwartz’s Portrait of Values Questionnaire [4, 48] (PVQ) comprises 57 brief descriptions (or portraits) of different individuals, with three descriptions for each of the 19 values. Each description portrays a person’s goals and aspirations, introduced with words such as ’It is important to him/her’, ’He/she thinks’ or ’He/she believes’. Participants were asked to indicate ’How much is this person like you?’ using a 6-point scale ranging from 1 (’Not like me at all’) to 6 (’Very much like me’). Cronbach’s alphas and McDonald’s omegas were above 0.79 for all measurements, except for humility (ranging between 0.46 and 0.64) and Power-Resources (ranging between 0.45 and 0.68). The 19 values can be grouped into four overarching dimensions: Openness to change (self-direction thought, self-direction action, stimulation, hedonism); Self-enhancement (achievement, power-dominance, power-resources); Conservation (humility, conformity-interpersonal, conformity-rules, tradition, security-personal, security-societal, face); Self-transcendence (universalism-tolerance, universalism-nature, universalism-concern, benevolence-caring, benevolence-dependability) [49].

#### COVID-19 health concern

We asked participants to rate the health concerns of the pandemic on a scale from 1 to 10 (with 1 for no concern and 10 for extreme concern).

### Analytical strategy

First, we examined the differences in values and well-being levels at T1, T2, and T3 with repeated measures ANOVAs. Second, we compared differences in well-being changes among men and women using repeated measures ANOVA. The within-subject factor was time (T1, T2, and T3), and the between-factor was gender. For all post hoc analyses, we used pairwise comparisons with the Bonferroni correction. Finally, we used hierarchical linear regression to test whether values before the pandemic (T1) were predictive of well-being during the pandemic (T2). The dependent variables were T2 indices of well-being. The controlled predictors at step one were age, gender, subjective health concern, and the T1 (pre-pandemic) baseline measure of given well-being facet. Predictors tested at step two were four pre-pandemic levels of higher-order indicators of human values (openness to change, self-enhancement, conservation, and self-transcendence). We used the minimum least square estimation method to calculate standardized parameters (β) and *R*^2^ change as the goodness of fit indicator. The α level of .05 was set. All statistical analyses were conducted in IBM SPSS Statistics for Windows, Version 26.0.

## Results

### Preliminary analysis

We found that the participants perceived the COVID-19 health concern as very high (*M* = 8.18; *SD* = 1.89). The relationship of health-related concern is a crucial factor with a potential impact on well-being during a pandemic. Thus we used it as a control variable in the analyses.

### Pandemic-related changes in values and well-being

Individuals increased valuing self-direction (thought), conformity (rules), humility, and universalism (nature and tolerance) across the first four weeks of the lockdown (T2 and T3 relative to T1) (Table 1). Two weeks into the lockdown, individuals increased valuing personal and social security, interpersonal conformity, caring (one of the benevolence dimensions), and universalistic concern. However, these increases diminished and were no longer significant four weeks into the lockdown. Finally, individuals decreased in valuing hedonism.

**Table 1:** Differences in values and well-being levels before and during the COVID-19 pandemic.

	Time			*F*	η^2^	Post hoc
	T1 *M(SD)*	T2 *M(SD)*	T3 *M(SD)*			
Self-direction—Thought	4.84(.73)	5.02(.68)	5.05(.67)	16.87***	.07	T1<T2, T3
Self-direction–Action	5.02(.71)	5.09(.68)	5.11(.67)	2.83	-	
Stimulation	3.53(.98)	3.58(1.06)	3.60(1.07)	1.14	-	
Hedonism	4.21(.90)	4.09(.96)	4.04(1.02)	7.36**	.03	T1>T2, T3
Achievements	3.82(.95)	3.84(.99)	3.92(.91)	2.30		
Power-dominance	2.44(1.04)	2.41(1.04)	2.41(1.10)	.17	-	
Power-resources	3.11(1.01)	3.16(1.00)	3.15(1.08)	.55	-	
Face	4.65(.85)	4.63(.91)	4.63(.87)	.10	-	
Security—Personal	4.85(.75)	4.96(.77)	4.89(.78)	3.93*	.02	T1<T2
Security—Social	5.00(.83)	5.11(.87)	5.05(.90)	3.29*	.02	T1<T2
Tradition	4.22(1.21)	4.20(1.28)	4.22(1.28)	.10	-	
Conformity—Rules	4.38(.92)	4.55(.95)	4.53(.91)	8.34***	.04	T1<T2, T3
Conformity–Interpers.	4.09(1.05)	4.25(1.07)	4.18(1.07)	5.81**	.03	T1<T2
Humility	4.11(.78)	4.25(.80)	4.26(.82)	5.83**	.03	T1<T2,T3
Benevolence—caring	4.98(.72)	5.09(.73)	5.03(.70)	3.46*	.02	T1<T2
Benevolence–depend.	5.03(.70)	5.06(.75)	5.04(.71)	.34	-	
Universalism—concern	4.85(.82)	4.95(.81)	4.88(.81)	3.24*	.02	T1<T2
Universalism—nature	4.63(.93)	4.74(.97)	4.72(.97)	3.44*	.02	T1<T2,T3
Universalism–toler.	4.65(.83)	4.78(.78)	4.76(.81)	5.64**	.03	T1<T2,T3
Satisfaction with life	3.92(1.09)	3.94(1.12)	3.77(1.14)	7.64**	.03	T1, T2>T3
Positive affect	3.01(.57)	2.88(.58)	2.82(.61)	17.32***	.08	T1>T2,T3
Negative affect	2.00(.72)	2.55(.72)	2.46(.74)	88.86***	.29	T1<T2>T3
Eudaimonic well-being	4.99(.72)	4.90(.73)	4.90(.68)	4.22*	.02	T1>T2, T2

*Note*. *F* based on Pillai’s trace with Greenhouse-Geisser correction; *Df* = 2, 428. T1 = nine months before the lockdown, T2 = two weeks into the lockdown, T3 = four weeks into the lockdown.

*p < .05

** *p* < .01

*** *p* < .001.

There was a decrease in well-being across all its facets. Individuals reported decreased satisfaction with life four weeks into the lockdown (at T3 relative to T1 and T2). Moreover, their eudaimonic well-being decreased from T1 to T2 (the third measurement did not differ from the other two). Finally, individuals experienced less positive affect and more negative affect after the lockdown relative to baseline. After its peak two weeks into the lockdown, negative affect decreased four weeks after the lockdown but did not return to pre-pandemic levels.

### Pandemic-related changes in well-being and gender

**Fig 1:**
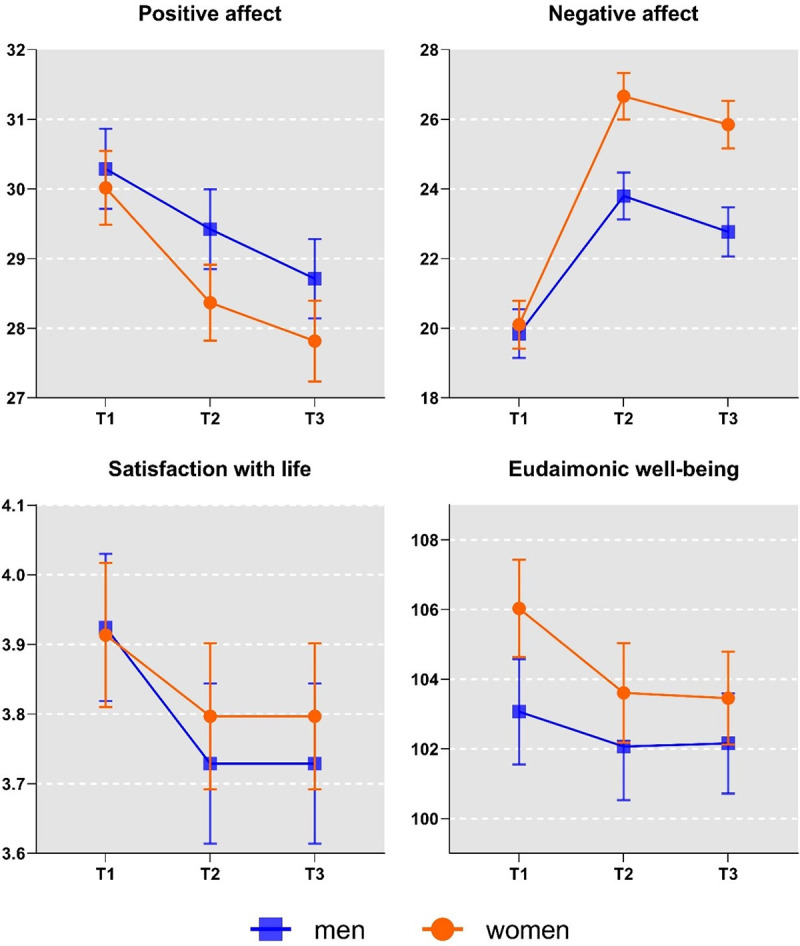
Changes in well-being and values mine months before, two weeks after, and four weeks after the lockdown. *Note*. T1 = nine months before the lockdown, T2 = two weeks into the lockdown, T3 = four weeks into the lockdown. Bars represent standard errors.

We observed that women responded to lockdown with stronger increases in negative affect than men (Fig 1 and Table 2). This effect was indicated by a significant interaction of time and gender in their influence on negative affect. In contrast, women and men had comparable levels of positive affect, satisfaction with life, and eudaimonic well-being.

**Table 2:** Main effects and interaction of univariate analyses.

variable	Measurement time		Gender		Interaction	
	*F*	η_p_^2^	*F*	η_p_^2^	*F*	η_p_^2^
Positive affect	15.86***	.07	1.09	-	.73	-
Negative affect	81.30***	.28	5.89*	.03	6.36**	.03
Satisfaction with life	7.78**	.04	.05	-	.33	-
Eudaimonic well-being	3.57*	.02	1.06	-	.73	-

*Note*. Gender coded as 1 = women, 2 = men.

* *p* < .05

** *p* < .01

*** *p* < .001

### Values as predictors of well-being changes

We found that individuals who were more open to change before the pandemic responded with higher levels of eudaimonic well-being two weeks into the lockdown (Table 3). This effect was significant after controlling for well-being baseline levels and control variables such as age, gender, and COVID-19 health concern. It also significantly increased the predictive power of the model for eudaimonic well-being. For explorative purposes, we also observed that older participants responded with more positive affect. Conversely, negative affect response was higher among individuals with high COVID-19 health concerns.

**Table 3:** Predictors of well-being during the first two weeks of the COVID-19 pandemic (T2).

Predictor	Satisfaction with life	Positive affect	Negative affect	Eudaimonic well-being
Step 1				
Well-being (T1)	.84***	.53***	.51***	.80***
Age	-.06	.13*	.03	-.04
Gender	-.01	.07	-.16**	.03
COVID-19 health concern (T2)	.02	.08	.31***	-.01
*F*	127.01***	23.97***	41.64***	85.73***
*R*^*2*^	.71***	.31***	.44***	.62***
Step 2				
Well-being (T1)	.82***	.49***	.48***	.72***
Age	-.05	.15*	.04	-.01
Gender	-.02	.07	-.19**	.04
COVID-19 health concern (T2)	.03	.08	.33***	-.02
Openness to change (T1)	.08	.11	-.14	.19**
Self-enhancement (T1)	.08	.03	.11	-.06
Conservation (T1)	-.10	.10	-.02	.05
Self-transcendence (T1)	.06	-.14	-.01	-.06
*F*	68.33***	12.92***	22.04***	45.99***
*ΔR*^*2*^	.02**	.02	.02	.02*

*Note*. Standardized regression coefficients (β). Gender coded as 1 = women, 2 = men. T1 = nine months before the lockdown, T2 = two weeks into the lockdown.

* *p* < .05

** *p* < .01

*** *p* < .001.

## Discussion

We examined the impact of the COVID-19 pandemic on how people evaluate their lives (well-being) and which pursuits they consider important in life (personal values). We used a baseline measurement from our ongoing project on a related subject. We asked its participants to report their well-being and values two and four weeks into the lockdown restrictions introduced in Poland. The participants reported very high levels of health concern related to the pandemic and its potential consequences. We found that the respondents experienced a considerable decrease in all aspects of subjective and eudaimonic well-being and that some of their values changed considerably. Valuing hedonism decreased, and some other values increased in importance (e.g., security, conformity, caring, universalism and self-direction). We also found gender differences as women responded with stronger negative affect to the lockdown. Finally, we observed that individuals who were more open to change before the pandemic achieved higher levels of eudaimonic well-being once the pandemic started.

The contribution of these findings is twofold. First, it presents how Poles responded to the pandemic—a finding that might be used in making informed decisions on public health policies and recommendations. Results from our national sample also contribute to the global evidence of the adverse impact of the COVID-19 pandemic on well-being and its transformative influence on other psychological dimensions [50]. Second, we present that values and well-being are meaningfully influenced by situational context, i.e., by the events and changes in life circumstances related to the COVID-19 pandemic. This is vital evidence that addresses the problem of stability and change in values [40, 51] and well-being [3, 10] and their relation to gender differences.

This study provides new evidence on how well-being reflects adverse external circumstances. We replicated and extended similar findings from other countries [24, 26–29, 52]. However, we were the first to present a decrease in eudaimonic well-being, an index of well-being related to self-expression [6]. Our findings add to the literature on stability and change in well-being. For instance, we found that each well-being measure decreased during the COVID-19 pandemic, but the dynamic was different for cognitive responses (satisfaction with life) than affect. For instance, satisfaction with life decreased after only four weeks of lockdown, but we did not observe such a decrease in its first two weeks. Contrastingly, individuals (women in particular) experienced the highest levels of negative affect immediately, two weeks into lockdown. This is in line with the subjective well-being model, where emotions are precursors to cognitive evaluations of life [53]. We also found that the strength of response in negative affect varied between genders. This effect might be explained by the personality theory of subjective well-being [11], which links well-being to stable traits such as neuroticism, which determine emotional reactions to adverse circumstances, e.g., the COVID-19 pandemic.

This is the first study to document how the COVID-19 pandemic influenced values relative to pre-pandemic levels. The problem with stability and change in values and the role of context in shaping individuals’ values has been discussed previously [40, 51]. However, our findings are the first to document that a global health concern is likely to produce a rapid reorganization in what people consider important in life. For instance, people seemed more eager to abandon hedonic pursuits during the lockdown. These findings correspond well with the notion of motivational hierarchy, i.e., when basic life resources seem to be threatened (also when lives are at stake), people are more likely to reduce the pursuits of pleasurable activities. This also suggests that the participants’ personal values reflected external pressure to reduce hedonic activities (e.g., outdoor physical training, attending concerts, going to the movies) and focus on self-protection. Participants’ values also adjusted to or reflected a need to nurture openness to change and protection. The increase of conservative pursuits (valuing security and conformity) corresponds well with previous findings that individuals are more prone to shift towards increasingly conservative values when the social or political context becomes less secure [40]. Finally, the pandemic also led to a rise in the importance of values oriented towards others as people became more eager to appreciate humility, benevolence, and universalism. These findings indicate that adverse global situations are likely to promote prosocial orientation [39]. It is noteworthy that some of the observed changes in values (e.g., prioritizing personal and social security) were transient, i.e., individuals returned to their baseline four weeks into the lockdown. This might suggest that the value system is flexible in that it bends but bounces back when the novel situation becomes more familiar.

Finally, we found that all the measures of well-being observed two weeks into COVID-19 lockdown were predicted mainly by their corresponding levels in the pre-pandemic measurement. This effect seems straightforward and is consistent with the adaptation level theory of well-being, which addresses the rank-order stability of well-being [10]. However, we also found that the pre-pandemic value dimension of openness to change was related to higher responses in eudaimonic well-being two weeks into lockdown. This new and exciting finding sheds light on how values are predictive of well-being and what functions they might serve. The present findings may be interpreted as supporting the values’ direct link [38] to well-being, i.e., individuals who value openness to change reacted to the COVID-19 pandemic more favorably. This might suggest that individuals high in Openness to Change may have used different interpretations of upcoming new events as an opportunity for self-exploration and self-expression, even if these events were as severe as a worldwide pandemic.

### Limitations

This study has several limitations. First, we used self-reports. Although we used measures with established validity in predicting real-life outcomes, our findings would be more robust if we had behavioral or observational data reflecting whether our participants’ beliefs and reported experiences translated into objective changes in their lifestyles. Second, our first baseline measure was taken nine months before the COVID-19 pandemic. A stronger approach would be to collect data closer to the pandemic, e.g., in December 2019, when few expected the emergence of this pandemic in Poland and other countries. Third, we covered only a small fraction of the pandemic. The analyses would provide much more information regarding the dynamic of values and well-being change if we collected more data waves before and after the pandemic. Fourth, we collected data in Poland. Thus, we cannot confirm whether these findings generalize to other countries with distinct dominant values and a different pandemic course. Finally, for obvious reasons, these data and the analytical models were correlational rather than experimental. Thus, our models do not provide direct evidence of causality. For instance, we cannot explain whether the effects that we observed were caused by the health concern, the economic threat, the lockdown, changes in work patterns and circumstances, social distancing, or a combination of these possible factors. Future studies might seek methods to dissect these effects as they are essential for making informed decisions on public policies and individual psychological help.

### Practical implications

This study has practical implications. First, we present data that might be used to guide public policies and recommendations for public health. For instance, we show that individuals seem to be ready to adjust their values to adverse contexts (at least in the initial phase of the pandemic). Thus, regulations that limit hedonistic pursuits are likely to be accepted by the general public. Furthermore, individuals presented a stronger need for prosociality, and these personal tendencies may be addressed by encouraging activism or voluntary work. Second, our findings seem adequate for evidence-based practice regarding psychological help during pandemics [54]. For instance, our participants seemed to have needed emotional support to buffer an intense negative response in the initial phase of the pandemic. Thus, practitioners should be fully aware that at least initially, the pandemic is likely to dominate the affective experience and that adverse cognitive responses are less likely to emerge initially but may do so later. Finally, we observed that individuals who were more open to change before the pandemic experienced greater eudaimonic well-being after the lockdown. This is a promising finding that might be further examined in an applied context, e.g., psychological interventions for promoting eudaimonic well-being in times of pandemic.

## Conclusions

**Fig 2:**
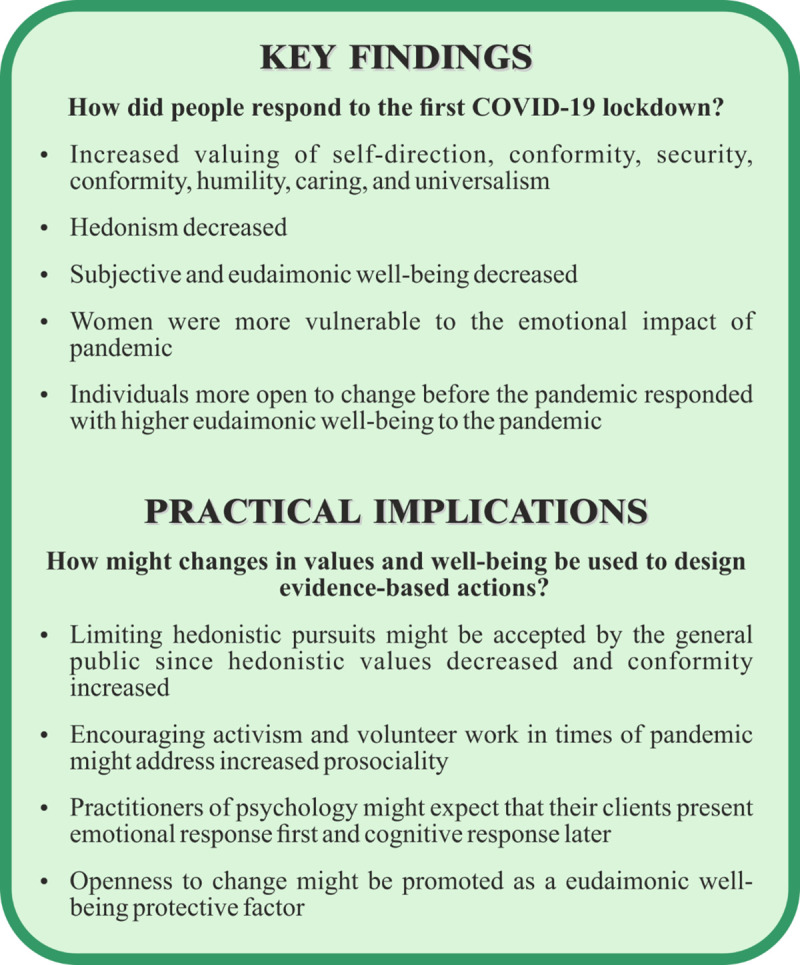
Summary of the findings and practical implications.

The COVID-19 pandemic has been a major global event with a strong psychological influence on individuals and communities [24, 26–29]. The strength of our project is that we provided novel data, replicated previous findings, and extended the scope of scientific analysis regarding the psychological impact of the COVID-19 pandemic on life. Consequently, we provide findings that may help better understand how individuals navigated the early phase of the COVID-19 pandemic. Fig 2 presents the summary of the findings and practical implications.
